# Mindfulness-Based Cognitive Therapy (MBCT) programme for depression in people with early stages of dementia: study protocol for a randomised controlled feasibility study

**DOI:** 10.1186/s40814-017-0143-x

**Published:** 2017-05-31

**Authors:** Elisa Aguirre, Josh Stott, Georgina Charlesworth, Deirdre Noone, Jacob Payne, Mina Patel, Aimee Spector

**Affiliations:** 1 Research and Development Department, North East London NHS Foundation Trust, Goodmayes Hospital, First floor, Maggie Lillie Suite, Ilford, IG3 8XJ UK; 2 Department of Clinical, Education and Health Psychology, University College London, London, UK

**Keywords:** Depression, Dementia, Alzheimer’s, MBCT, Feasibility outcomes, RCT

## Abstract

**Background:**

Depression and dementia are major public health problems in the UK. Depression in early-stage dementia is very common and significantly reduces quality of life, speeds cognitive decline and increases functional impairment. Mindfulness-Based Cognitive Therapy (MBCT) is an effective depression prevention programme, and the National Institute for Clinical Excellence (NICE) has suggested that MBCT is a priority for implementation. Alongside this, there is emerging evidence demonstrating promising results in relation to the benefits of adapted mindfulness interventions for people with dementia, suggesting that it could be beneficial in reducing depressive symptoms and in slowing deterioration in cognitive functions such as sustained attention, distraction inhibition and task switching.

**Methods:**

The design is a single-blind randomised controlled feasibility trial. Participants with mild to moderate depression and early stages of dementia will be recruited from the participating memory services. Participants will receive either immediate or delayed access to an 8-week MBCT programme. Participants will be assessed by a blind assessor and complete cognitive and mood-related outcome measures before and after the intervention. This feasibility study will test the trial design and assess recruitment, retention, acceptability and adherence, as well as providing preliminary efficacy data.

**Discussion:**

This study will inform the design and sample size for a future full randomised controlled trial (RCT), which will be carried out to determine the effectiveness of the intervention in reducing depressive symptoms in people with early stages of dementia.

**Trial registration:**

ClinicalTrials.gov ISRCTN16382776

## Background

Depression and dementia are major widespread public health problems in the UK, with the numbers of people with dementia set to rise to 1.7 million by 2051 [1]. It is estimated that 50% of people with dementia experience some symptoms of depression, and the rate of persistence of symptoms is high [2]. Evidence suggests that the onset of depression is linked to a dementia diagnosis [3]. Comorbid depression in dementia (i) significantly reduces quality of life [4], (ii) speeds cognitive decline [5], (iii) increases functional impairment and the need for nursing home placements [6], (iv) is associated with higher mortality rates [7], (vi) increases carer stress [8] and (vii) is costly for health resources, with an increased use of expensive inpatient beds [9].

The Improving Access to Psychological Therapies (IAPT) programme has successfully rolled out services providing Cognitive Behavioural Therapy (CBT) in England [10], with good outcomes in terms of depressive symptom reduction [11]. The government has now pledged to increase access to evidence-based therapies to all patient groups, including older people and people with dementia [12]. However, there is currently little evidence for the benefit of psychological interventions for depression in people with dementia [6]. A recent meta-analysis has concluded that Mindfulness-Based Cognitive Therapy (MBCT) is an effective depression prevention programme for people without dementia, but with a history of recurrent depression [13]. In addition, the National Institute for Clinical Excellence (NICE) has suggested that MBCT is a priority for implementation. Alongside this, there is emerging evidence demonstrating promising results in relation to the benefits of adapted mindfulness interventions for people with dementia, suggesting that it could be beneficial in reducing depressive symptoms and in slowing deterioration in cognitive functions such as sustained attention, distraction inhibition and task switching [14].

Recently, evidence from an adapted mindfulness intervention feasibility study for people with dementia in care homes (*n* = 31) showed that a modified mindfulness intervention was feasible and that it led to significant improvements in quality of life [15]. There were no significant changes in mood. However, the study did not actively recruit people with depression and a third of the participants from the intervention group who were in the clinically depressed range at baseline moved out of the range at follow-up. In contrast, none of the participants in the control arm did, suggesting that the intervention should be further tailored and target people with comorbid depression and dementia.

This current study will be the first to investigate the feasibility and potential efficacy data of MBCT in people with comorbid dementia and depression, using qualitative and quantitative methods as recommended by the Medical Research Council [16].

The aim of this study is to provide evidence for the feasibility of conducting a fully powered RCT to evaluate a complex psychological intervention for depression in people with early stages of dementia. Feasibility will be evaluated in it in terms of rates of recruitment, retention and adherence to treatment protocol. The potential efficacy of the intervention will also be estimated.

## Methods/design

### Design

A single-blind randomised feasibility trial will be carried out to test the delivery of an MBCT intervention for people with mild to moderate depression and comorbid early stages of dementia. In this RCT, 50% of participants will be randomly allocated to the immediate group (IA) and will receive MBCT immediately. The remaining 50% of participants will be allocated to the delayed access control (DAC) group. Both arms will receive treatment as usual. Participants will be assessed by blinded assessors that will not be involved in the delivery of the intervention at any point. Assessors will provide explicit reminders to the participants before the assessment visit so that participants do not disclose group allocation inadvertently. The assessor will also record their impression of which arm of the trial the participants belong to and their confidence in that prediction on completion of the follow-up assessments. This will enable us to assess whether inadvertent loss of blinding leads to bias.

Alongside the running of the study, a progress evaluation will be carried out by interviewing participants taking part in the groups in order to gather qualitative information that can support and enhance the quantitative findings of the feasibility study. In order to gather in-depth data regarding people with dementia’s experience of attending a mindfulness group, participants that attended the group will be invited to a semi-structured interview after the group. Approximately 10–12 interviews with group participants and family caregivers will be conducted. Carers of participants will be invited to further examine whether potential benefits of the MBCT intervention were generalised to other areas that relate to activities of daily living such as engagement in home-based practice and improvements observed at home.

### Study setting and location

Participants will be recruited and identified from memory services in North East London NHS Foundation Trust and memory services in Oxleas Foundation Trust.

### Target group and sample size

As this is a feasibility pilot trial and there is limited research on the effects of mindfulness-based interventions for depression for people with dementia, it was difficult to estimate the likely effect size. We took a sample of 32 participants, 16 per group, as this was considered feasible for the requirements of this pilot trial [17].

#### Inclusion criteria

People meeting the inclusion criteria are those treated for or with a diagnosis of mild depression and a diagnosis of mild to moderate dementia according to DSM-IV criteria with a Mini Mental State Examination (MMSE) [18] of 18 or above.

#### Exclusion criteria

People meeting the exclusion criteria are those who (1) have congenital learning disability; (2) present with severe depression or high risk of self-harm (e.g. suicidal intent) requiring urgent intervention; (3) are within 2 months of bereavement; (4) are involved in other psychosocial intervention research; or (5) have a diagnosis of psychosis.

### Intervention

The MBCT treatment protocol will be adapted from MBCT for the prevention of depression relapse [19]. The main adaptation will be the substitution of longer length of meditation practices with shorter ones, discussion of patient’s mood and cognitive symptoms in-session and encouraging participants to use formal mindfulness exercises as well as informal mindfulness when distressing situations arise during the week. We will also shorten the length of the mindfulness meditation for home practice from 45 to 15–20 min and will increase attention to distress from memory problems during in-session and at-home exercises. The adapted MBCT will consist of eight, weekly 1 h and half group sessions, which will include skills training and in-class practice. Specific in-class mindfulness exercises will include (a) ‘mindful eating’ (the ‘raisin exercise’), (b) ‘body scan’ exercise, (c) ‘mindful stretching’ and (d) sitting ‘mindfulness’ meditation exercises with various objects (breath, body, sounds, emotional states, thoughts). The programme will incorporate daily assignments of ‘formal’ home practice of mindfulness techniques (using 15–20-min audio-recordings as well as ‘informal’ exercises to integrate mindfulness into everyday experiences (e.g. eating, walking and brushing teeth). Participants will be instructed to practise mindfulness exercises aided by audio-recordings at least 5 days a week, as well as practising mindfulness throughout the day (e.g. while walking, eating, showering), for an additional 10–15 min a day, for a total of 25–40 min of total practice per day. Patients will be ask to record daily practice times in homework logs which will be collected each week, in which they will check which audio-recording(s) they had listened to that day and how much time they had spent doing other mindfulness practice throughout the day. A weekly telephone call by a member of the team will aim to support home practice.

### Procedure for recruitment and intervention delivery

The researchers will present information about the study (rationale, inclusion and exclusion criteria, etc.) to the managers of the memory clinics (six in total). They will then discuss the feasibility of running the study in their service (e.g. available transport for participant, expected attrition rate for groups in the service), and if they agree to take part, they will start identifying suitable participants for the study.

Participants will then be informed of the study by their clinical leads from the different memory services that have agreed to take part in the study, and after expressing an interest in the study, potential participants will be asked to complete eligibility pack which will include a participant information sheet along with a consent form. Participants will have the opportunity to ask questions about the study by phone, email or post before being asked to complete the consent form. Until eligibility has been assessed and informed consent has been given, individuals will not be able to begin participation in the study.

People with dementia meeting the inclusion criteria and who consent to take part in the study, giving signed informed consent in accordance with the provisions of the Mental Capacity Act 2005 will be included in the study. Clinical psychologist trainees that will be involved in the process will receive informed consent and Mental Capacity Act training. Where the participant’s level of impairment increases, so that they were no longer able to provide informed consent, the provisions of the Mental Capacity Act 2005 will be followed. The initial giving of informed consent will provide an indication of the person’s preference for participation in the research, and the family caregiver’s viewpoint will also be sought. If the person with dementia shows discomfort at any point with the assessments or their participation in the groups, they will be discontinued.

Included participants will complete baseline assessment and be randomised into either the IA and will receive MBCT immediately or the DAC group. Randomisation will take place remotely by an independent member of the Research and Development Department using the online software sealedenvelop.com. Taking up or declining the study will not affect the usual support and treatment they receive.

The two clinical researchers aim to run two MBCT groups each, two at each site with approximately eight participants in each. The clinicians running the groups will monitor the feasibility of recruiting participants (for both IA and DAC groups) within the given time frame. This will ideally include 16 participants in each of the two locations.

At least two clinicians have been allocated to each of the groups so that in the event that a group facilitator is unavailable (e.g. leave, sickness), the remaining facilitator will run the group alongside co-facilitators.

The qualitative interviews with participants and their carers will be conducted within 3 weeks of the groups having been completed. As such, it is expected that all data collection will have been completed by December 2016.

### Treatment as usual

Usual care will be the same for both groups and involve usual GP and memory service support, anti-depressant medication, old age psychiatry review and community psychiatric nurse appointments. For both groups, we will record the care received to enable a full description of treatment as usual.

### Outcome measures

The primary outcome measures will assess feasibility outcomes and be:

- Recruitment rate (incorporating willingness of patients to be randomised and practicality of the MBCT intervention)
  The recruitment will be deemed feasible if it is possible to recruit the required 32 participants in the given time frame of 4 months (June 2016–September 2016).
- Retention rate (incorporating acceptability of the therapy)
  The attrition and retention rates will be assessed and defined as the number of sessions completed and people assessed at follow-up, including details of withdrawals. We will judge an acceptable completion rate as 55% of people completing four or more of the eight course sessions and a follow-up completion rate of at least 75%.
- Incidence of adverse events
  We will be collecting details of any adverse events that might arouse from evaluations and or delivery of the intervention.
- Acceptability of the developed MBCT programme
  We will be using qualitative interviews in order to further assess the acceptability and perceptions of the users in relation to the intervention, and its results will inform the final design of the intervention and consider this intervention as acceptable for this client group.
- Acceptability of the clinical outcome measures for inclusion in the study
  We will be assessing the feasibility of the outcome measures by exploring the missing data from the measures used and examine the inclusion exclusion rates from the recruitment process, and this will inform future RCT trial design.

The secondary outcome measures will assess patient- and carer-centred outcome measures and be conducted by the assessor in a private room in the memory clinic or in the participant’s home. The following measures will be completed before and after the intervention (around 3 months) and 6-month post intervention.

- Cornell Scale for Depression in Dementia (CSDD) [20]
  Depression will be assessed using the CSDD [20], a 19-item clinician-administered instrument. Each item has a 3-point scale (0 = absent, 1 = mild or intermittent, 2 = severe) with scores ranging from 0–38. The clinical cut-off for significant depressive symptoms is a score of 8 and above [18].
- Patient Health Questionnaire (PHQ-9) [21]
  PHQ-9 is a freely available mood rating questionnaire consisting of nine questions mirroring DSM-IV depression diagnostic criteria, and each rated 0–3 giving a maximum score of 27. Cut-off scores are used to label depression severity as 0–4, minimal depression; 5–9, mild depression; 10–14, moderate depression; 15–19, moderately severe depression; and 20–27, severe depression.
- Quality of Life–Alzheimer’s Disease Scale: Participant Version (QoL-AD) [22]
  Quality of life will be measured using the Quality of Life–Alzheimer’s Disease (QoL-AD) scale [22], a 13-item self-report questionnaire. Information about several areas was gathered from the PWD and their carer, such as physical health, mood, friends, fun, self and general life. The measure has excellent internal consistency and inter-rater reliability. The content, criterion and construct validity are good [23].
- Cognitive and Affective Mindfulness Scale (CAMS-R)
  The Cognitive Affective Mindfulness Scale (Revised) [24] is a 12-item self-report questionnaire that measures trait mindfulness in day-to-day experience. Scores range from 0 to 48, with higher scores indicating higher levels of mindfulness. The measure was used in the aforementioned pilot study [25].
- Generalised Anxiety Disorder 7-item (GAD-7) scale [26]
  The GAD-7 is a 7-item questionnaire focusing on symptoms of anxiety experienced in the past 2 weeks. Each item is rated according to the frequency of the described problem.
- Rating Anxiety in Dementia (RAID) [27]
  Anxiety will be assessed using the RAID scale [27], an 18-item clinician-administered instrument. Each item is rated on a 4-point scale (0 = absent, 1 = mild or intermittent, 2 = moderate, 3 = severe) with scores ranging from 0 to 54. The clinical cut-off for significant clinical anxiety was a score of 11 and above. The RAID scale is the most appropriate measure for assessing anxiety in PWD [28].

### Weekly measures

PHQ-9, GAD-7 and CSDD will be collected after each mindfulness session, in line with the service protocol.

### Carer measures

This will be conducted by the assessor in a private room in the memory clinic or in the participant’s home. The assessor will ask a member of staff or family member questions about the relevant participant’s, mood, anxiety and quality of life. The information obtained will be used in combination with the information gathered from the interview with the participant to complete the following assessments:

- Cornell Scale for Depression in Dementia [20]
- Quality of Life–Alzheimer’s Disease Scale: Carer Version [22]
- Rating Anxiety in Dementia (carer version) [27]

### Statistical analyses

Baseline characteristics will be summarised for all participants, the IA and DAC groups separately. Participant uptake of and adherence to the intervention, defined as the number of sessions completed and the number of support sessions participants take part in, as well as data follow-up rates, will be summarised and presented as percentages. To assess potential efficacy in each group separately, data will be entered into SPSS and analyses will be conducted using SPSS statistics for Windows (Version 24; IBM Corp.). Although determining differences in clinical outcomes between the three arms is not the primary purpose of this trial, comparisons will be undertaken to investigate the feasibility of studying these outcomes and to calculate estimates for the likely effect sizes and 95% confidence intervals. As recommended in guidelines for good practice for the analysis of pilot studies [29], the focus of the results will be on the estimates of the treatment effects rather than on statistical significance, and as such, no hypothesis testing will be undertaken. The qualitative interviews will be analysed qualitatively using thematic analysis [30] to identify emerging themes from within the data.

Finally, a power calculation using Gpower 3 [31] will be carried out to determine the required sample size for a future RCT.

### Ethical considerations

Ethical approval has been granted by the HRA London City and East REC, with REC approval number 16/LO/0578.

#### Confidentiality

Data collected by the research team will be kept either on fingerprint or password-locked memory sticks, on fingerprint identification laptops, stored in a locked filing cabinet in the researcher’s office on the site of University College London or will be saved on a secured network space that only the researchers have access to. Personally, identifying information will be removed from the main electronic data files for analysis by the researchers. A coded file with sensitive data will be stored separately.

## Discussion

### Importance of the project

Depression and early dementia tend to present at the same time and are major widespread public health problems in the UK. However, currently, there is no evidence available for a beneficial treatment that can be offered to this population. This novel project will determine important factor imperative in the consideration of a future RCT design such as recruitment, adherence, acceptability and follow-up rates of the MBCT group for depression in people with early stages of dementia. We hope that recruiting and identifying people with dementia from memory services that present with mild to moderate levels of depression who might otherwise fail to engage in traditional NHS-based primary psychological services for depression will help to address the wider treatment gap in postdiagnostic services for people with dementia. The design allows a detailed description of those recruited, in order to understand the age, current and past treatments and severity and chronicity of depression amongst those recruited.

If this study shows that the trial design is feasible, then, this will lead to a larger, funded RCT of the intervention, which may have important implications for supporting people with early stages of dementia and create a pathway for postdiagnostic interventions for dementia.

### Benefits to participants

Participation within this research study will allow people with early stages of dementia presenting mild to moderate depression to participate and engage in an MBCT programme aimed at reducing depression. Research has provided us with empirical evidence that the proposed approach is effective in aiding a variety of problems related to depression in those without dementia and that it might be a useful approach for people with early stages of dementia.

Results from this research will be used to indicate whether the intervention is an acceptable resource for people with early stages of dementia and comorbid depression. By assessing the acceptance and feasibility of the intervention for this population, we will be able to gain a key insight as to how such an intervention can be best delivered to individuals and details in relation to how to implement it within the NHS.

In collaboration with Oxford Mindfulness Centre, public engagement and knowledge exchange in different populations, and their expert clinicians and researchers, will be able to assist our intervention clinicians in case they may need additional help. They are fully committed to helping with this study, and they have granted the study team to access their intervention materials for this feasibility study. Working with OMC at this early feasibility stage will lay a foundation for the future large RCT which, if it is successful, will provide support for the evaluation and introduction of MBCT in the NHS as an additional psychological intervention to offer to people with early stages of dementia and depression and a viable option for depression postdiagnosis.

### Dissemination

Dissemination will include an executive summary, open access journal publications and conference presentations. The main journal publication will present the study findings with the data being used to inform the development of the full RCT protocol. A simple feedback sheet information in relation to the results of the study will be sent to all participants who have taken part. The findings will also be disseminated to the Oxford Mindfulness Centre in the form of a report and an oral presentation. Oxford Mindfulness Centre will then disseminate the results internally. We will also disseminate findings via the newsletters on the widely used www.omc.com website.

## Abbreviations

DAC: Delayed access control
IA: Immediate access
MBCT: Mindfulness-Based Cognitive Therapy
RCT: Randomised controlled trial
